# A 49-Year-Old Woman Presenting With Cavitary Lung Lesions: A Case Report

**DOI:** 10.7759/cureus.25219

**Published:** 2022-05-22

**Authors:** Sonali Khurana, Rajeeva Raju, Paul M Shaniuk

**Affiliations:** 1 Internal Medicine, University Hospitals Cleveland Medical Center, Cleveland, USA; 2 Internal Medicine, Case Western Reserve University School of Medicine, Cleveland, USA; 3 Pathology, Louis Stokes Cleveland Veterans Affairs Medical Center/Case Western Reserve University School of Medicine, Cleveland, USA; 4 Internal Medicine, Louis Stokes Cleveland Veterans Affairs Medical Center/Case Western Reserve University School of Medicine, Cleveland, USA

**Keywords:** case report, granulomatosis with polyangiitis, anti-neutrophil cytoplasmic antibody, vasculitis, cavitary lung lesions

## Abstract

Granulomatosis with polyangiitis (GPA) can be a challenging diagnosis to confirm due to significant overlap with other small-vessel vasculitis syndromes and similar presentations to non-vasculitic processes such as infection or malignancy. We report a case of a 49-year-old woman presenting with several months of cough, chest wall pain, and weight loss, who was found to have several cavitary lung lesions on imaging, no renal involvement, and unusual anti-neutrophil cytoplasmic antibody (ANCA) serologies. After tissue biopsy, the patient was diagnosed with GPA and treated with steroids and rituximab with clinical improvement at follow-up. Due to a complex clinical course and non-classic symptomatology, her diagnosis of GPA was not discovered until several months after symptom onset. Clinicians should consider GPA in the case of progressive cavitary lung lesions even in the absence of renal involvement or positive ANCA serologies, as prompt tissue diagnosis is crucial to allow for early initiation of treatment.

## Introduction

Granulomatosis with polyangiitis (GPA) is a small-vessel vasculitis associated with anti-neutrophil cytoplasmic antibodies (ANCA) in approximately 90% of cases [1]. Patients may present with non-specific constitutional symptoms, or they may have the more classic organ-specific involvement such as nasal or oral inflammation, abnormal chest imaging, or abnormal urinary sediment [1]. Due to a significant overlap between the various small-vessel vasculitides and a highly variable clinical presentation, standardizing an approach to classify and diagnose GPA is complex and challenging [1,2]. In this report, we present a case where a patient presented with non-classic clinical symptoms of GPA and unusual ANCA serologies, which ultimately led to a delayed diagnosis.

## Case presentation

A 49-year-old woman presented to the emergency department (ED) with two months of progressive cough, pleuritic left chest wall pain, night sweats, and dyspnea. There was associated fatigue, unintentional 20-pound weight loss, and occasional trace hemoptysis in the week prior to this presentation. She also noted an intermittent, photosensitive rash on her lower extremities and face and chronic joint pain in her shoulders, elbows, knees, and ankles with morning stiffness lasting approximately 10 minutes and improving with activity. Past medical history was notable for recurrent sinusitis, type 1 diabetes mellitus, Hashimoto thyroiditis, fatty liver disease, microscopic colitis, seizure disorder, conversion disorder, and fibromyalgia. She had a remote smoking history of a half pack of cigarettes per day for five years. She reported remote exposure to the trialed anthrax vaccine but had no known tuberculosis exposure.

On arrival at the ED, the patient was afebrile, with a heart rate of 104 beats/minute, blood pressure of 119/78 mmHg, respiratory rate of 14 breaths/minute, and oxygen saturation of 94% on room air. She was in no apparent distress and breathing comfortably. On examination of the head, eyes, ears, nose, and mouth, she was noted to have mild conjunctival pallor and bilateral telangiectasias over the face with nasolabial sparing. A cardiac exam showed tachycardia but regular rhythm without murmurs, and lungs were overall clear to auscultation. Abdominal, extremity, musculoskeletal, dermatologic, and neurologic exams were unremarkable.

Preliminary investigation revealed mild leukocytosis with a white blood cell count of 12,100/microliter, with a differential of 47.6% neutrophils, 38% lymphocytes, 11.6% monocytes, 2.1% eosinophils, and 0.7% basophils, a hemoglobin level of 13.3 grams per deciliter (g/dL), and platelet count of 177,000/microliter. Electrolytes and renal function were normal. Urinalysis was without protein, blood, or casts. There was a mild elevation in alkaline phosphatase to 155 international units per liter (IU/L), and albumin was 2.6 g/dL.

**Figure 1:**
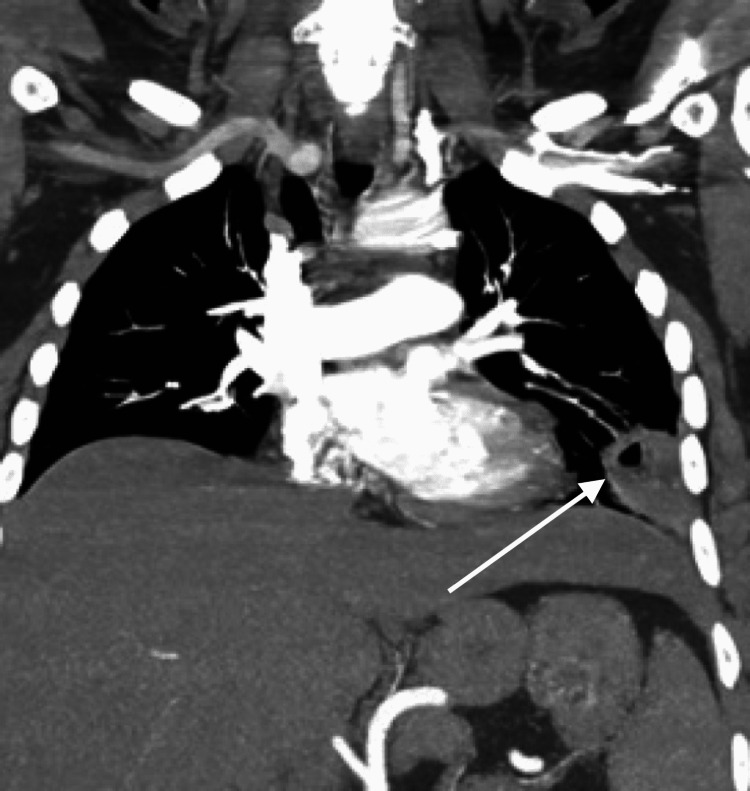
Chest CT (coronal view) demonstrating left lower lobe cavitary lesion.

**Figure 2:**
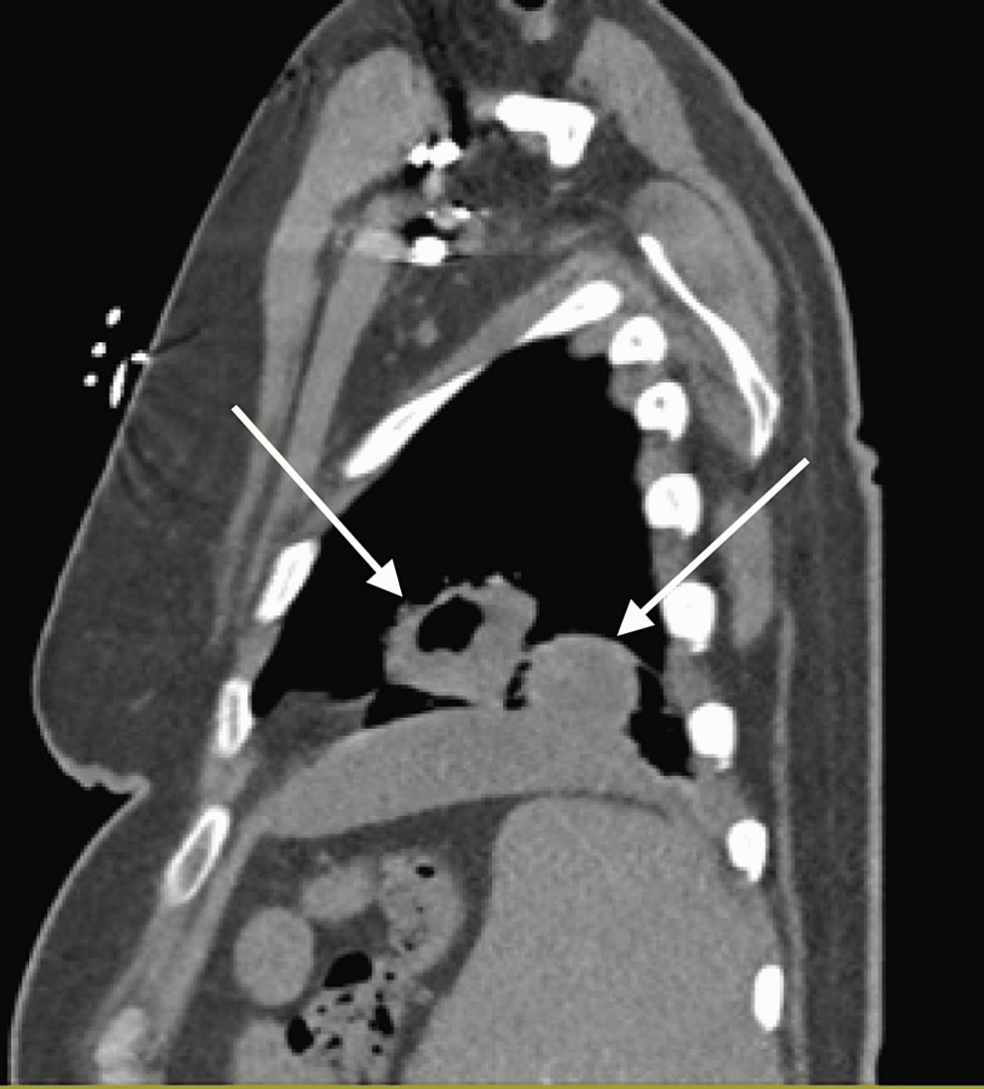
Chest CT (sagittal view) demonstrating two left lower lobe cavitary lesions.

Computed tomography (CT) imaging of her chest demonstrated two cavitary masses (4.3 x 3.0 cm and 3.6 x 2.5 cm) in the left lower lobe and lingula, respectively, and also showed patchy nodular airspace disease within the dependent aspect of bilateral lower lobes, and a small layering left pleural effusion (Figures 1, 2).

Acid-fast bacillus testing was negative. Multiple sets of blood cultures were negative, but sputum culture grew methicillin-sensitive *Staphylococcus aureus*. Transthoracic echocardiogram was negative for infective endocarditis. The initial working diagnosis was lung abscess versus cavitary pneumonia, and the patient was treated with antibiotic therapy and ultimately discharged. However, she did not demonstrate complete resolution of symptoms and was ultimately admitted three more times for worsening of similar symptoms and was treated with increasingly broad-spectrum antimicrobials and discharged.

**Figure 3:**
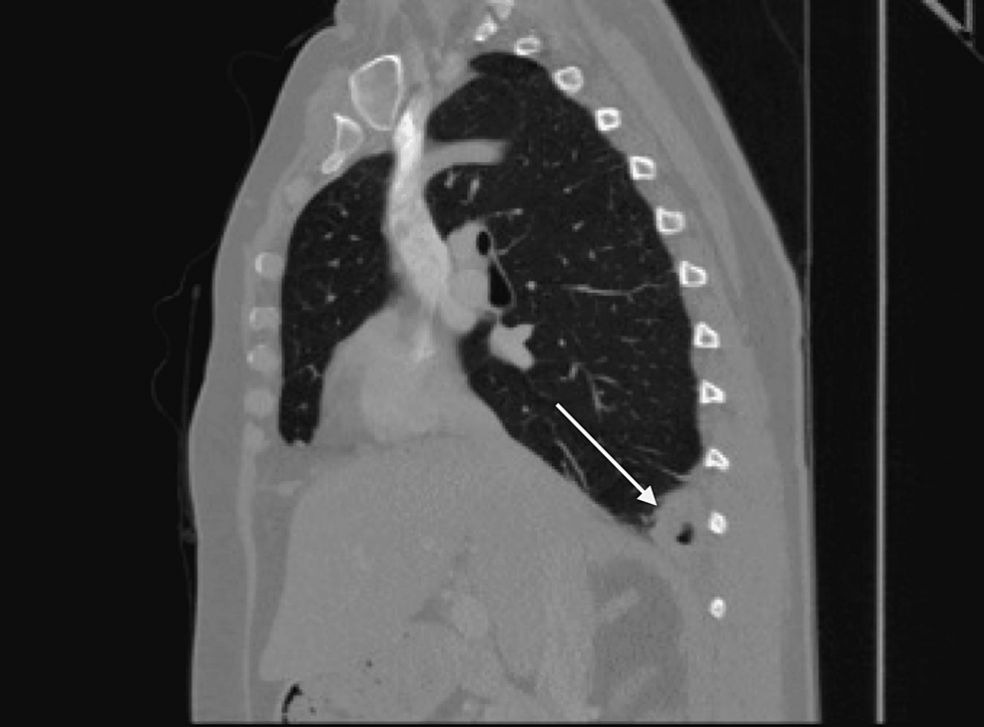
Chest CT (sagittal view) demonstrating right basilar cavitary lesion.

She presented again six weeks after the initial presentation, now complaining of right-sided chest pain. Repeat CT of the chest revealed a slight increase in the size of the prior two cavitary lesions with small left-sided pleural effusion, in addition to a new 3.7 x 2.4 cm spiculated right lower lobe cavitary lesion (Figure 3).

All three cavitary lesions were fluorodeoxyglucose (FDG)-avid on positron emission tomography (PET). She underwent thoracentesis for her small pleural effusion. Pleural fluid was exudative by Light's criteria, but pathology demonstrated inflammatory cells only, without malignant cells, and cultures were negative. CT-guided lung biopsy of left lung cavitary lesion showed fragments of fibrous tissue with necrosis. Further workup demonstrated a positive anti-nuclear antibody (ANA) titer of 1:320 in a homogeneous pattern, ANCAs were negative for both cytoplasmic anti-antineutrophil cytoplasmic antibodies (c-ANCA) and perinuclear anti-antineutrophil cytoplasmic antibodies (p-ANCA), but anti-proteinase 3 antibody was positive at 6.2 units per milliliter (U/ml; normal range: 0.0-3.5 U/ml). Of note, the anti-proteinase 3 antibody was repeated three weeks later at the request of the rheumatology team and had increased to 11.2 U/ml.

**Figure 4:**
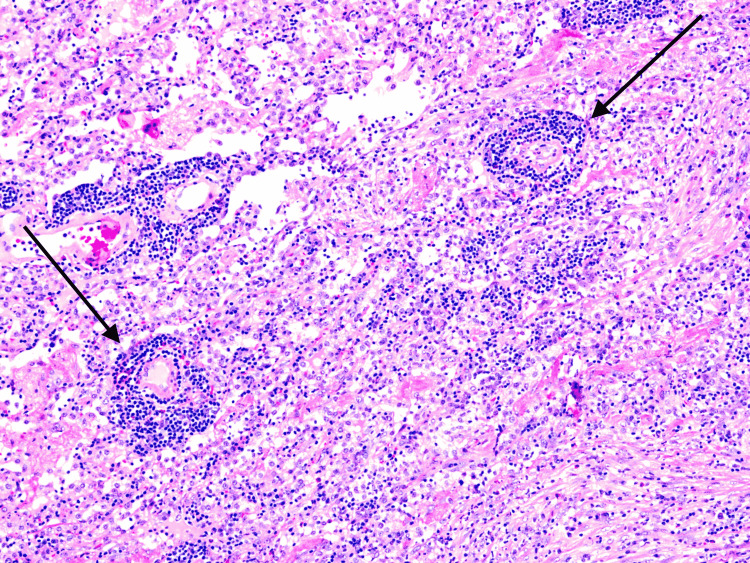
Lung biopsy (hematoxylin and eosin stain, 100x magnification). Small to medium-size blood vessels showing prominent perivascular cuffing by lymphocytes (vasculitis).

Ultimately, this patient underwent right-sided video-assisted thoracoscopic surgery with right-lower lobe wedge resection. Biopsy results from this tissue revealed acute and necrotizing granulomatous inflammation most consistent with GPA (Figure 4).

The patient was started on 40 milligrams (mg) of prednisone daily with 375 milligrams per square meter (mg/m2) intravenous rituximab infusions weekly for four weeks, and continued 30 mg of prednisone daily after completion of rituximab. The patient reported tolerating these therapies well and reported adherence to daily prednisone. Incidentally, she developed appendicitis one month after initiation of rituximab, which was unanticipated and thought to be unrelated to her GPA or treatment modalities. Repeat anti-proteinase 3 antibody at that time was undetectable. Her admissions for chest pain and GPA-related symptoms ceased and the prednisone was tapered over one month to her current maintenance dose of 10 mg daily. She did not keep her appointment for a repeat CT of the chest, so the size of lesions could not be monitored in response to the above therapy.

## Discussion

GPA is a vasculitis syndrome with a highly variable clinical presentation. The key features to identify include nasal or oral inflammation, abnormal chest imaging (varying from nodules to fixed infiltrates to cavitary lesions), abnormal urinary sediment (microscopic hematuria with or without red cell casts), and granulomatous inflammation on biopsy of an artery or perivascular area [1]. Though these features are not unique to GPA, they are commonly seen in this syndrome and are helpful in the diagnostic process. Organ involvement varies considerably in each patient with tracheal and pulmonary disease occurring in a majority of patients, ear, nose, and throat manifestation occurring in about 90% of cases, and renal involvement occurring in less than 20% of patients at initial presentation but later developing in up to 85% of cases within the first two years [2]. Cutaneous, neurologic, gastrointestinal, and many other organ-specific manifestations have been described but are less common [2]. This patient had pulmonary disease consistent with GPA, but the absence of nasal and renal involvement or typical serology findings made it difficult to confirm this diagnosis in real time.

The mainstay of diagnosis for GPA is a biopsy. The decision to pursue biopsy is often based on high suspicion for vasculitis given clinical manifestations and laboratory investigation, and most importantly ANCA testing. ANCAs are autoantibodies that target and attack specific proteins within neutrophils, with a common target in GPA being proteinase 3 [3,4]. While a positive ANCA strongly suggests vasculitis, approximately 10% of patients with GPA will be ANCA-negative [5]. In these cases, targeting specific proteins such as the proteinase 3 may help guide the diagnosis especially when the pre-test probably of GPA is high. Both false positives and false negatives occur with ANCA testing, so these results should be considered within each specific clinical context and should be correlated with biopsy findings [5,6]. This patient’s mildly positive anti-proteinase 3 antibody was suggestive of vasculitis; however, the negative ANCA serologies and other non-classic features kept the differential diagnosis broad. Infection was very high on the differential, but her symptoms relapsed despite broader-spectrum antimicrobial treatment, and thus she required a repeat biopsy to clarify the cause and type of inflammatory syndrome.

Biopsy in GPA can demonstrate a range of acute and chronic inflammation. Inflammatory histologic features such as the presence of neutrophils, eosinophils, macrophages, vasculitis, and necrosis have led to questions of appropriate diagnosis; however, data demonstrate that these features are seen in a statistically significant portion of patients with GPA as compared to patients without GPA [7]. In this patient, a biopsy of the new cavitary lesions was most consistent with GPA, thus allowing initiation of appropriate treatment with good clinical response.

Prompt diagnosis is essential to permit the initiation of treatment, which can often be organ-sparing and even life-saving. The therapy approach in GPA involves induction with glucocorticoid therapy in combination with either rituximab or cyclophosphamide [8]. Patients require close monitoring during induction and maintenance therapy, and occasionally require repeat imaging to ensure resolution of active disease. They tend to do very well with this therapeutic approach, often resulting in disease remission with a low risk of relapse. This patient continued to report ongoing fatigue and some pain secondary to chest lesions after initiation of steroid and rituximab therapy, but her dyspnea had improved significantly, and her hospitalizations ceased. Though repeat imaging was recommended upon completion of rituximab infusions, she continued to miss appointments for chest CT re-imaging and ultimately transitioned her care to a different local hospital system, but at her last follow-up visit, several months after discharge, she was doing well. The rheumatology team has since weaned her prednisone with success, and she has not kept follow-up appointments with rheumatology.

## Conclusions

In conclusion, GPA can present with a wide spectrum of symptoms. Patient presentation may carry significant overlap with other small-vessel vasculitis syndromes, infectious processes, and malignancy. It may also lack the classic renal or sinus involvement, as in this case, thus making GPA a difficult diagnosis to confirm. Providers often pursue ANCA testing to aid in the diagnosis of GPA; however, both false positives and false negatives can occur with ANCA testing. Thus, results of ANCA testing should be carefully considered within each specific clinical context in combination with testing for specific proteins (such as anti-proteinase 3 antibody) and with tissue biopsy.

Tissue biopsy is critical if clinical suspicion for GPA is high despite negative ANCA serologies as single organ involvement occurs not infrequently and is not required to make the diagnosis. Clinicians should consider GPA in the case of progressive cavitary lung lesions even in the absence of renal involvement or positive ANCA serologies, as prompt tissue diagnosis is crucial to allow for early initiation of treatment. Close monitoring and follow-up with treatment are also imperative, especially in a non-classic presentation, to ensure the resolution of the disease and further verify the diagnosis of GPA.
